# Identification and Classification of Long Non-Coding RNAs in the Mammary Gland of the Holstein Cow

**DOI:** 10.3390/ijms241713585

**Published:** 2023-09-01

**Authors:** Sahar Ghulam Mohyuddin, Yan Liang, Yuxin Xia, Mengqi Wang, Huimin Zhang, Mingxun Li, Zhangping Yang, Niel A. Karrow, Yongjiang Mao

**Affiliations:** 1 Key Laboratory for Animal Genetics, Breeding, Reproduction and Molecular Design of Jiangsu Province, College of Animal Science and Technology, Yangzhou University, Yangzhou 225009, China; saharbutt-18@hotmail.com (S.G.M.);; 2 Joint International Research Laboratory of Agriculture and Agri-Product Safety of Ministry of Education of China, Yangzhou University, Yangzhou 225009, China; 3 Department of Animal Science, Laval University, Québec, QC G1V-0A6, Canada; 4 Center for Genetic Improvement of Livestock, Department of Animal Biosciences, University of Guelph, Guelph, ON N1G-2W1, Canada

**Keywords:** long non-coding RNAs, mammary gland, Chinese Holstein cows, RNA sequence analysis

## Abstract

The mammary glands, responsible for milk secretion, are regulated at a local level by various hormones, growth factors, non-coding RNAs, and other elements. Recent research has discovered the presence of lncRNAs in these glands, with suggestions that they may be essential for the maintenance and function of mammary glands. Besides directly controlling the gene and protein expression, lncRNAs are believed to play a significant part in numerous physiological and pathological processes. This study focused on examining the mammary gland tissues of Chinese Holstein cows, to identify and categorize long non-coding RNAs (lncRNAs). The research intended to distinguish lncRNAs in the mammary tissues of Holstein cows and contrast them between lactation and non-lactation periods. In this study, mammary gland tissues were sampled from three Holstein cows in early lactation (*n* = 3, 30 days postpartum) and non-lactation (*n* = 3, 315 days postpartum) on a large dairy farm in Jiangsu province. Mammary tissue samples were collected during early lactation and again during non-lactation. In total, we detected 1905 lncRNAs, with 57.3% being 500 bp and 612 intronic lncRNAs. The exon count for lncRNAs varied from 2 to 10. It was observed that 96 lncRNA expressions markedly differed between the two stages, with 83 genes being upregulated and 53 downregulated. Enrichment analysis results revealed that Gene Ontology (GO) analysis was primarily abundant in cellular processes. The Kyoto Encyclopedia of Genes and Genomes (KEGG) pathway analysis indicated that target genes were predominantly abundant in metabolic pathways, fatty acid biosynthesis, the immune system, and glycosphingolipid biosynthesis. This study analyzed the expression profile and characteristics of lncRNAs in the mammary gland tissues of Holstein cows during both lactation and non-lactation stages, forming a foundation for further investigation into the functional roles of lncRNAs in Holstein cows throughout lactation.

## 1. Introduction

As a source of biologically active components, milk plays an important role in the growth and development of mammalian infants, including the development of their immune system, intestinal system, nervous system, and even intellectual development [1]. The mammary gland is responsible for secreting milk for infant nutrition [2,3]. It is essential that milk production and the growth and development of the calves are dependent on the supply of milk by healthy mammary glands in dairy cows. The mammary glands can, however, become infected with different pathogens and develop inflammation (mastitis). Using genomics [4,5], transcriptomic [6], and proteomics [7], mastitis has been extensively studied owing to its influence on dairy production. Factors such as genetics, epigenetics, and the environment affect dairy cow lactation and pregnancy. An important part of lactation is the development of mammary glands and the production and secretion of milk. Mammary gland development has been studied extensively in recent years, particularly protein-coding genes and microRNAs (miRNAs). Molecular biology studies have shown that non-coding RNA (ncRNAs) may play a significant role in mastitis [8]. In addition to viruses, bacteria, fungi, and mycoplasma [9,10], bovine mastitis can also be associated with more than 100 pathogens [11,12]. Bacteria such as Staphylococcus, Streptococcus, and Gram-negative bacteria are the most frequent causes of mastitis [13,14,15,16].

There is a wide variety of ncRNA, with long ncRNA (lncRNA) being one class that plays regulatory roles in a wide range of biological activities [17,18]. LncRNA transcripts are >200 nucleotides in length and constitute the majority of ncRNA transcripts in mammals [19], which regulate cellular expression directly [20]. It has been previously published that cis-lncRNA and trans-lncRNA are involved in gene regulation [21]. Additionally, lncRNA can stimulate or inhibit transcription, regulate protein activity, or modulate chromatin function [22,23,24]. Among the ncRNA, LncRNAs are most similar to mRNAs, especially in terms of their biogenesis pathways and formation. RNA polymerase II transcribes most lncRNAs, which have methylguanosine caps (5′) and are frequently spliced and polyadenylated [25]. There are alternative pathways for non-polyadenylated lncRNAs that arise either through RNA polymerase III promoters [26,27] or from the splicing and production of small nucleolar RNA [28]. There are also different ways in which lncRNAs are regulated during their biogenesis, maturation, and decay [18]. In cattle, approximately 23,000 lncRNA transcripts have been reported [29]. There are different numbers of lncRNAs in tissues [30,31,32] and different methods of identifying them based on RNA sequencing [33]. As one of the first studies to characterize lncRNAs in bovine mammary glands, Koufariotis et al. [34] reported that using polyA(+) captured transcripts from RNA sequencing across 18 tissue samples; he describes a list of lncRNAs. He discovered 9778 class 3 transcripts in total, of which at least one tissue sample had moderate-to-high expression in all three replicates and no protein-coding potential [34].

LncRNAs are important participants in gene regulation because they interact with coding genes both locally (cis) and distantly (trans) [35,36]. As the number of whole transcriptome sequencing studies (WTSS) continues to increase, there is a growing demand to prioritize single nucleotide polymorphisms from diverse datasets. The challenge in identifying and inferring the causal mutations related to clinical mastitis was emphasized in [37]. In the context of mammary gland development, it has been established that lncRNAs play critical roles as regulatory elements. Despite being relatively less explored in cattle, there have been significant advancements in the identification of lncRNA transcripts. Huang et al. conducted a study and reported the discovery of 449 lncRNAs, which are located in 405 intergenic regions, marking an important milestone as the first genome-wide catalog of intergenic lncRNAs in bovine species. These findings contribute to our understanding of the complex regulatory mechanisms underlying mammary gland development in cattle [38]. Approximately 3746 differentially expressed lncRNAs were identified from the dry and lactation mammary glands of Holstein cows and lncRNAs have been found to have growth inhibitory effects in mammary epithelial cells [39].

In cattle, the mammary gland samples of Chinese Holstein cows were analyzed to identify the RNA sequence, lncRNA, and protein-coding genes. They identified 1000 putative lncRNAs, 117 of which were differentially expressed between peak- and late-lactation stages. Overall, the 117 lncRNAs and 254 protein-coding genes were differentially expressed in the bovine mammary gland between peak- and late-lactation stages [40], whereas lncRNA related to mammary gland development of cows is still unrevealed. As a result of the identification of gene-wide lncRNAs involved in cattle mammary gland development and lactation, a molecular network regulating lactation will be constructed. Furthermore, key functional genes in immunity can be identified, which can serve as a basis for developing molecular breeding programs for high-quality cattle. As lncRNAs play an important role in gene expression in mammary gland tissue, high-throughput RNA sequencing (RNA-seq) was used in this study to identify differentially expressed lncRNA profiles during early lactation and non-lactation in Holstein cows. LncRNAs, especially during transcription, have remained a research hotspot in recent years. The aim of the study was to identify and categorize lncRNAs in Chinese Holstein cow mammary gland tissues. The mammary gland tissues of Chinese Holstein cows were the main subject of this study. The goal of this study was to identify and classify the lncRNAs found in the tissues of the mammary glands in order to better understand their potential roles in the development and production of milk in the bovine mammary glands. By understanding the functions of these lnc, we can develop new strategies for improving milk production and cow health in the future.

## 2. Results

### 2.1. Characterization and Identification of Long Non-Coding RNAs

**Figure 1:**
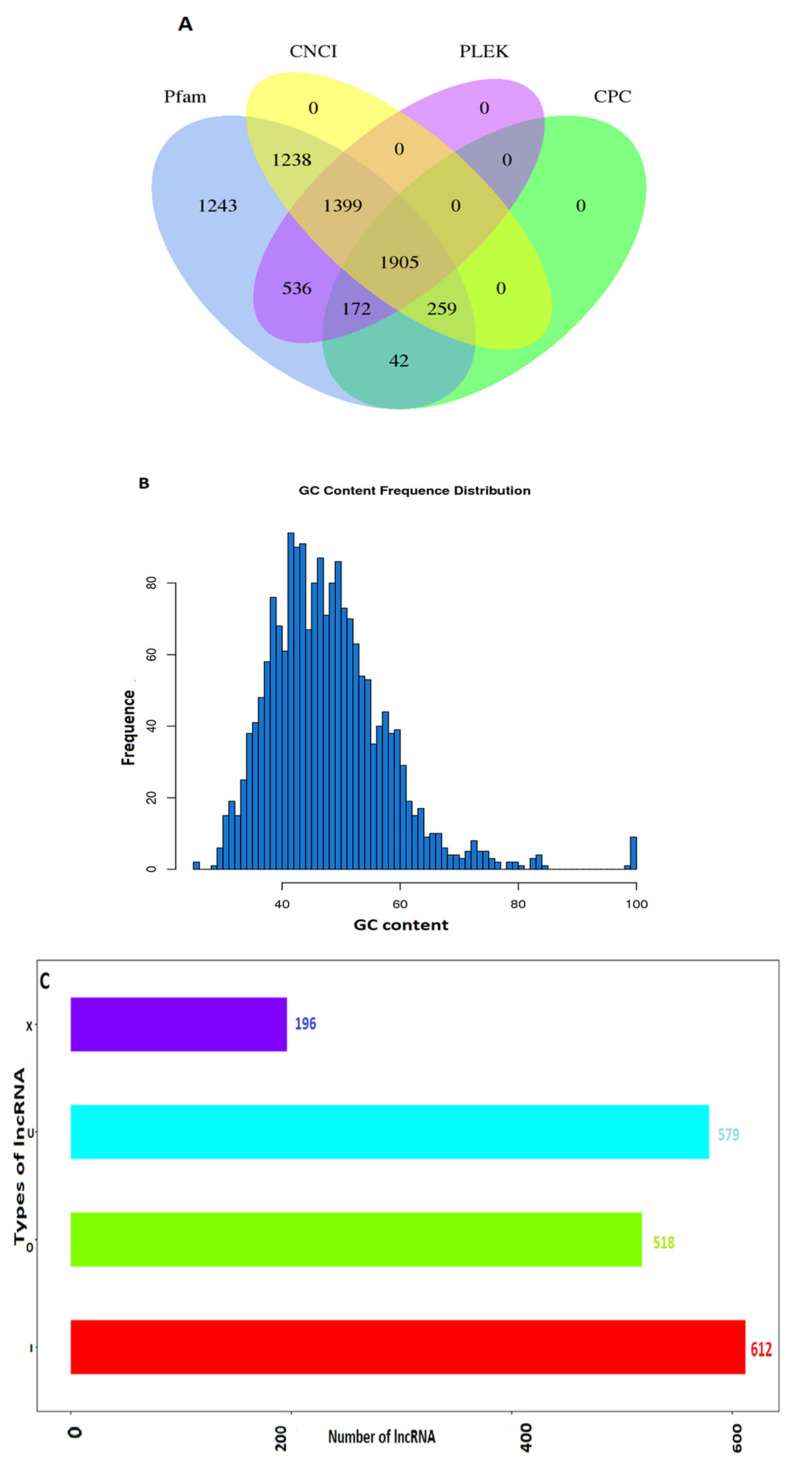

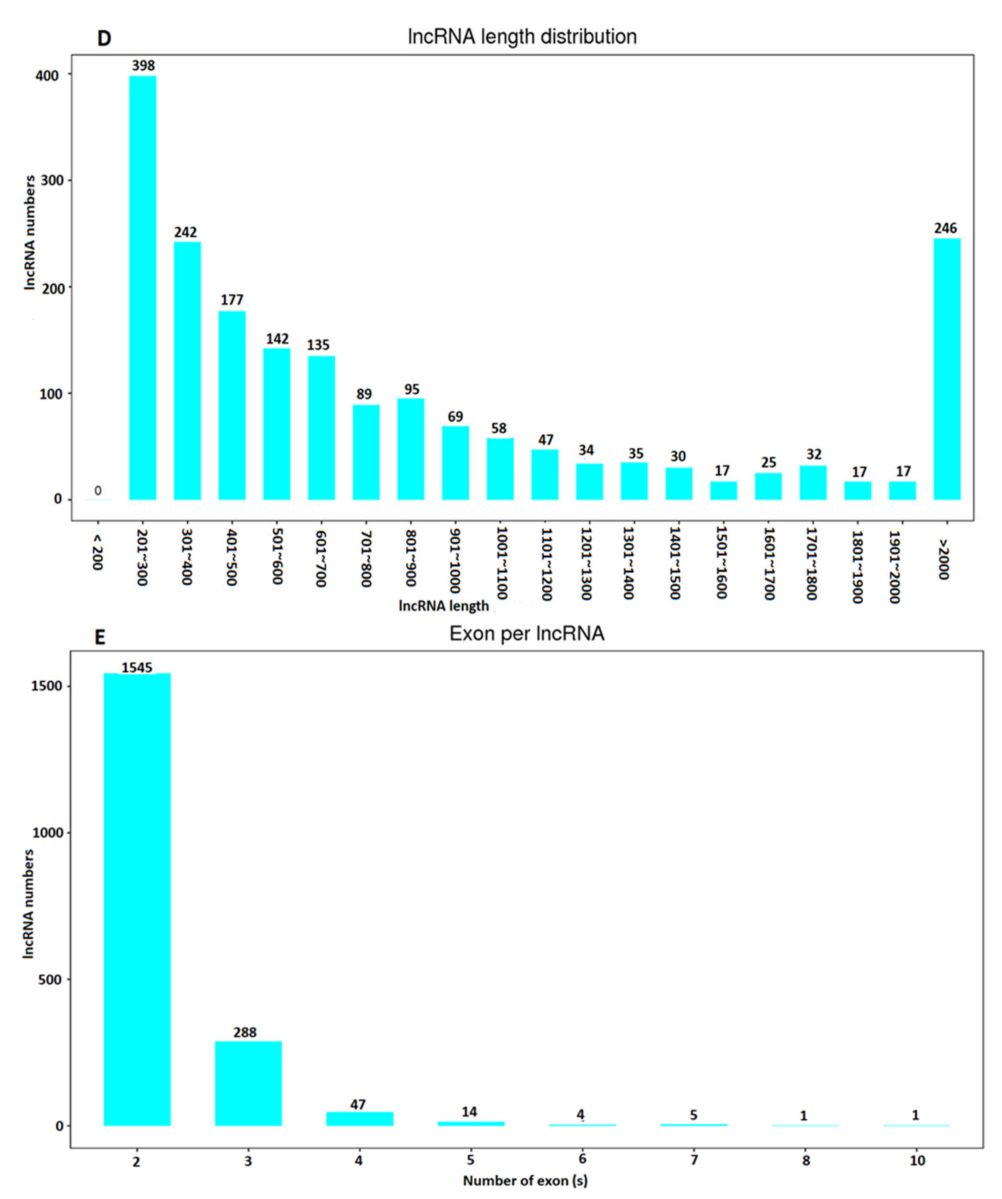
Identification, characterization, and types of lncRNAs. (**A**) Candidate lncRNA: Venn diagram showing coding capacity prediction results. A Venn diagram showing the lncRNA transcripts generated by Pfam, CNCI, PLEK, and CPC. The lncRNA identified by all four software types was used for downstream analyses. (**B**) Distribution map of lncRNA GC content. The vertical axis is the number of lncRNAs, and the horizontal axis is the value of lncRNA GC content. (**C**) LncRNA type statistical map. (**D**) LncRNA sequence length distribution map. The vertical axis is the number of lncRNAs, and the horizontal axis is the lncRNA length range. (**E**) Distribution of lncRNA exon number.

According to the characteristics of lncRNAs, lncRNA transcripts were predicted by CPC (Coding Potential Calculator), CNCI (Coding-Non-Coding Index), (protein family) Pfam, and PLEK respectively, and a total of 1905 lncRNAs were screened, as shown in Figure 1A. Headings for Table 1: (1) All: total number of lncRNAs; (2) ≥200 bp: length ≥200 bp number of lncRNAs; (3) ≥500 bp: length ≥500 bp number of lncRNAs; (4) ≥1000 bp: length ≥1000 bp number of lncRNAs; (5) N50: add the sequences in order from long to short; when the added length reaches half of the total length of the sequence, the last added sequence length is N50; (6) Total Length: the total number of bases of lncRNA; (7) Max Length: the longest lncRNA length; (8) Min Length: the shortest lncRNA length; (9) Average Length: Average length. Statistics on the distribution of the newly predicted lncRNA sequence length are shown in the following Figure 1D. The final predicted lncRNA sequence statistical information after screening is shown in Table 1. The distribution of GC content for the newly predicted lncRNA sequences is shown in Figure 1B. According to the characteristics of genome mapping, most 612 lncRNAs are located in intronic regions, while 196, 518, and 579 lncRNAs are antisense, sense-overlapping and intergenic, respectively (Figure 1C). According to the position of the known protein-coding transcript, lncRNA can be divided into the following four types: intergenic lncRNA (character u), intronic lncRNA (character (i), anti-sense lncRNA (character x), and sense-overlapping lncRNA (character o). The four types of statistics for newly predicted lncRNA are shown in the following (Figure 1C). The transcript length of identified lncRNAs ranged from 201 to 18,449 nucleotides, with 70.6% of lncRNAs shorter than 1000 nucleotides (Figure 1D). The number of exons contained with lncRNAs ranged from 2 to 10, and these were concentrated between 2 and 3. The vertical axis is the number of lncRNAs, and the horizontal axis is the number of exons contained in lncRNAs (Figure 1E).

### 2.2. Analysis of Different lncRNA Expression

**Figure 2:**
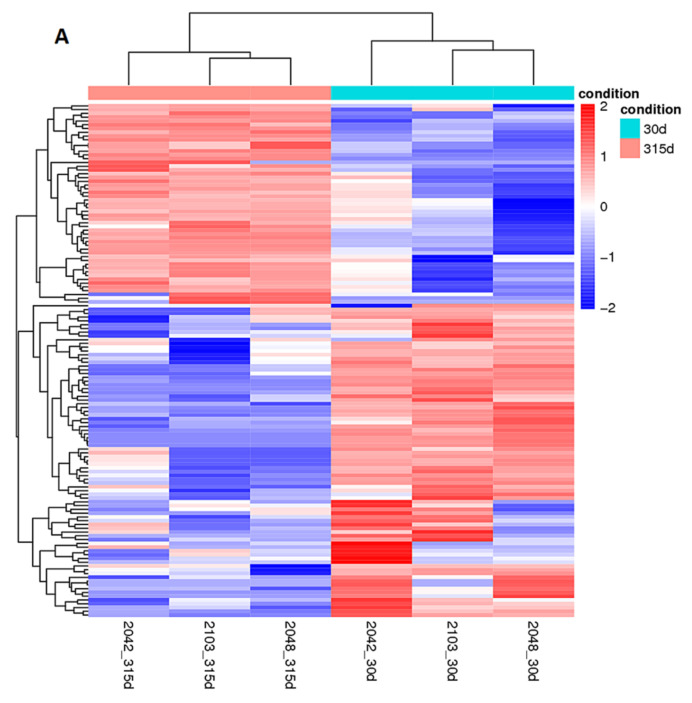

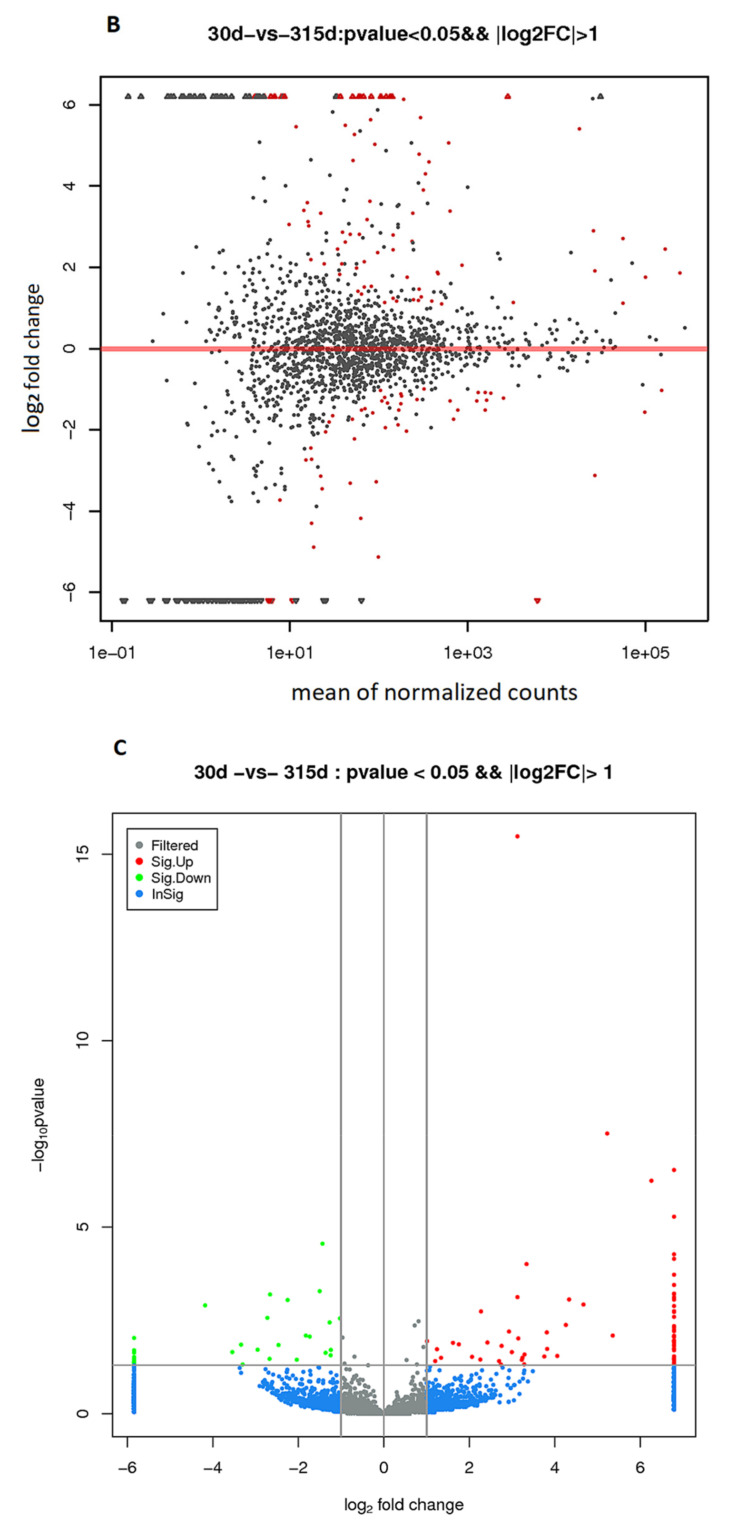
Differentially expressed lncRNAs in mammary gland tissue from Holstein cows before calving and early lactation. (**A**) Cluster analysis results map. (**B**) Differential expression lncRNA MA map (**C**) Differential expression lncRNA volcano map (gray and blue being lncRNAs with non-significant differences, and red and green being lncRNAs with significant differences, with no changes can be observed in blue dots).

RNAseq data revealed the comparison of the early-lactation 30 days postpartum and non-lactation 315 days postpartum. A total of 136 differentially expressed lncRNAs were found in the 315 day non-lactation group, of which 83 were upregulated and 53 were downregulated. Similarly, Table 2 represents the differentially expressed some lncRNA transcripts among the 1905 candidate lncRNA transcripts (42 were upregulated and 18 were downregulated). For the differentially expressed lncRNAs, unsupervised hierarchical clustering was used. Clustering allows the same class of samples to appear in the same cluster, and genes clustered in the same cluster may have similar biological functions. The cluster analysis results can be seen in Figure 2A. The difference caused by the comparison is reflected in the MA plot, (M (log ratio) and A (mean average) scales); the *X*-axis is the average of all sample expressions used for comparison after standardization, and the *Y*-axis is log2FoldChange; distinctive genes marked in red are significant (by differential screening) (Figure 2B). The overall distribution of differentially expressed lncRNAs can be understood by plotting volcano plots. The differences generated by the comparison are reflected in the volcano plot, with gray and blue being lncRNAs with non-significant differences, and red and green being lncRNAs with significant differences. The *X*-axis is the display of log2 FoldChange, and the *Y*-axis direction is the display of log10 Pvalue (Figure 2C). The gene with no changes can be observed in blue dots. Some differentially expressed up- and downregulated transcripts can be obtained from Table 2.

### 2.3. Differential Expression of Long Non-Coding RNA Genes in Holstein Cows during Lactation

**Figure 3:**
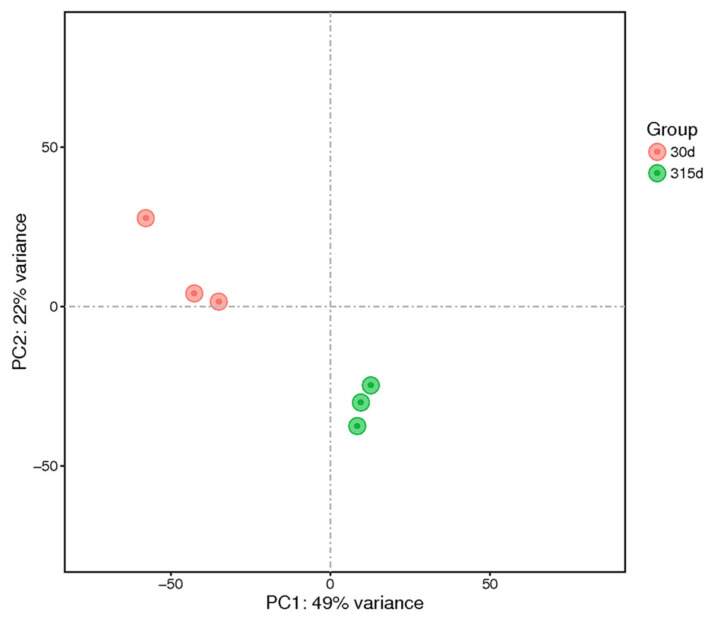
Principal component analysis (PCA) plot of the samples from 2 lactation periods.

Understanding the mammary gland function of Holstein cows is dependent on unraveling the intricate web of functional genes that govern this process and their underlying biological networks. To investigate this complication, a thorough investigation involving principal component analysis was conducted, encompassing all protein-coding and lncRNAs genes. The findings revealed an astonishing segregation of the two distinct groups under consideration: early lactation (*n* = 3, 30 days postpartum), and non-lactation (*n* = 3, 315 days postpartum). As shown in Figure 3, these groups were compellingly divided into well-defined clusters. Furthermore, when sorted by the number of days, these samples provide an excellent opportunity to investigate differentially expressed genes. PCA can show the relationship between samples in different dimensions. The closer the sample clustering distance or PCA distance, the more similar the samples; the different groups of samples are distributed in different areas of two-dimensional or three-dimensional space, and the samples of the same group are more concentrated in space. The PCA diagram is shown in the following figure. Researchers can embark on a comprehensive exploration of the molecular dynamics that drive Holstein cow mammary gland development by exploiting the power of these data, thereby unraveling the critical mechanisms that underscore this intricate process.

### 2.4. Differentially Expressed lncRNA and mRNA Co-Expression Analysis

**Figure 4:**
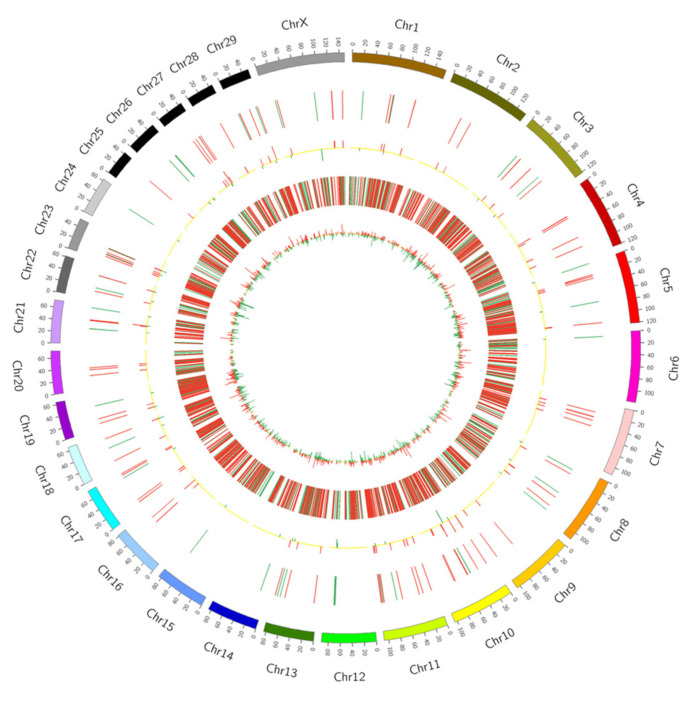
Circos plot of differentially expressed lncRNAs and mRNAs.

**Figure 5:**
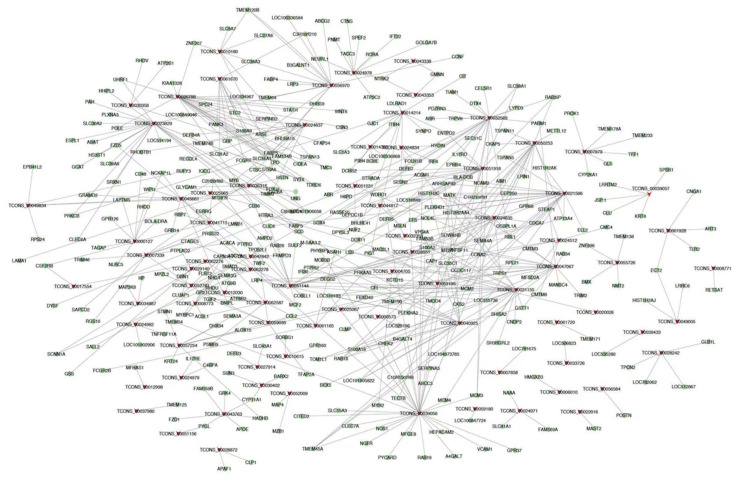
lncRNA and mRNA co-expression network diagram. Note: red arrow nodes indicate differentially expressed lncRNAs, and green circle nodes indicate differentially expressed mRNAs.

Co-expression analysis of differentially expressed lncRNAs and mRNAs entails examining gene expression patterns between lncRNAs and protein-coding messenger RNAs (mRNAs) that exhibit significant changes in expression levels. This study contributes to the understanding of the complex interplay between lncRNAs and mRNAs, shedding light on their potential roles in various biological processes and pathways. We used Circos software (v0.67) to visually represent the information about differentially expressed lncRNAs and mRNAs, as shown in Figure 4. The outermost circle represents the chromosomal distribution of the species under study, schematically. The second circle depicts the distribution of differentially expressed lncRNAs across the chromosomes, with red and green lines representing upregulated and downregulated lncRNAs, respectively. The third circle displays a bar chart illustrating the abundance of differentially expressed lncRNAs at different genomic locations, with higher columns indicating a greater number of differentially expressed genes. The fourth circle, which uses the same color scheme as the lncRNAs, represents the distribution of differentially expressed mRNAs along the chromosomes. The innermost circle contains a bar chart that depicts the expression patterns of differentially expressed mRNAs at various genomic positions, mirroring the color distribution seen in the lncRNA analysis. In addition, we used Cytoscape (Version: 3.7.2) to create a co-expression network diagram for the top 500 differentially expressed lncRNAs and mRNAs, as shown in Figure 5. This network visualization provides an in-depth look at the potential interactions and relationships between these differentially expressed lncRNAs and mRNAs, shedding light on the underlying regulatory mechanisms and functional associations. We improve our understanding of the complex interplay between lncRNAs and mRNAs by utilizing these visualization techniques, allowing us to identify key players and pathways involved in the observed differential expression patterns. These findings pave the way for additional research into the functional significance of the identified lncRNAs, as well as their potential roles in modulating gene expression and cellular processes.

### 2.5. Differentially Expressed lncRNA Target Genes and Functional Analysis

**Figure 6:**
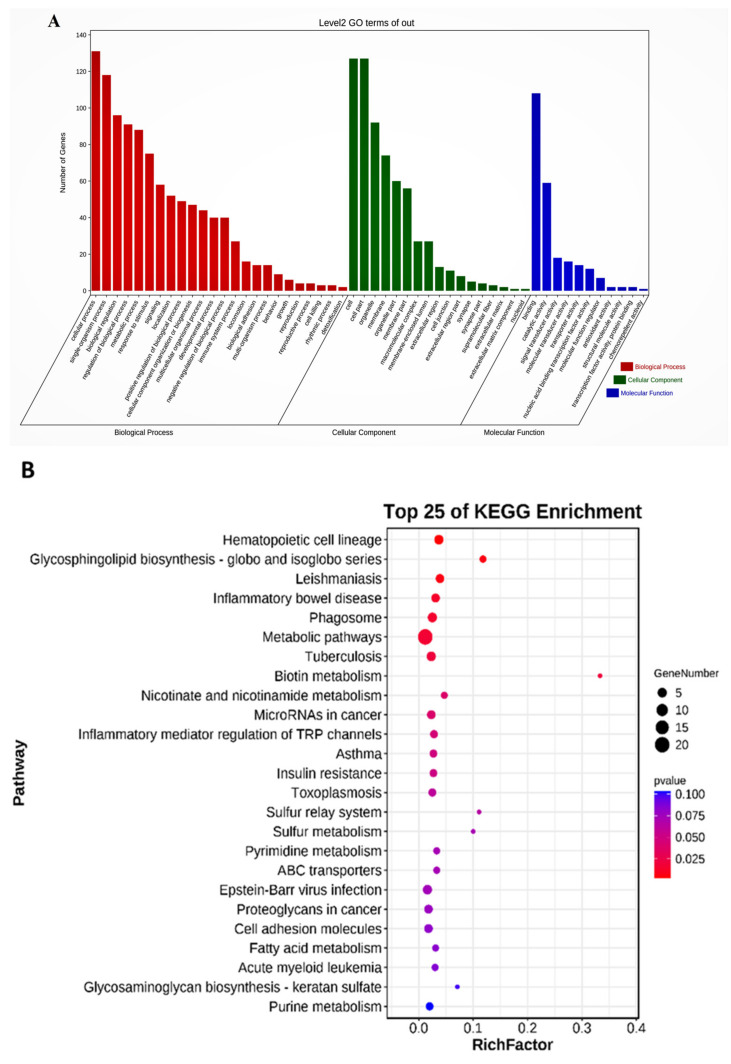
Functional analysis of lncRNA target genes. (**A**) GO enrichment analysis of differentially expressed lncRNA target genes. (**B**) KEGG pathway analysis of differentially expressed lncRNAs target genes.

We conducted an analysis to identify their target genes in both cis and trans to gain insight into the functional roles of the differentially expressed lncRNAs. Gene Ontology (GO) and Kyoto Encyclopedia of Genes and Genomes (KEGG) pathway analyses were performed on the identified target genes. The results of the GO analysis are depicted in Figure 6A, which provides a comprehensive overview of the biological processes, cellular components, and molecular functions associated with the target genes. The target genes were primarily involved in developmental processes, biological regulation, biological process regulation, single-organism processes, metabolic processes, and immune system processes. This suggests that the differentially expressed lncRNAs are involved in a variety of biological activities, such as growth, regulation, and immune responses. The analysis of cellular components revealed that the target genes were mostly found in extracellular regions, extracellular matrix components, and cell junctions. These findings indicate that they play a role in intercellular communication, tissue structure, and cell adhesion. In terms of molecular functions, the target genes were linked to nucleic acid-binding transcription factor activity, binding, and structural molecule activity. This suggests that they may play a role in transcriptional regulation, molecular interactions, and the maintenance of cellular structure and integrity. The KEGG pathway analysis (Figure 6B) also revealed the enriched pathways associated with the target genes. The target genes were discovered to be involved in metabolic pathways, hematopoietic cell lineage pathways, fatty acid biosynthesis, immune system processes, and glycosphingolipid biosynthesis, among other things. These findings imply that the differentially expressed lncRNAs and their target genes may be involved in vital biological processes such as energy metabolism, blood cell development, immune responses, and lipid metabolism. By identifying their target genes and providing insights into their roles in various biological processes and pathways, these comprehensive analyses shed light on the functional implications of the differentially expressed lncRNAs. These findings pave the way for future research into the molecular mechanisms underlying the regulatory functions of lncRNAs in the context studied.

## 3. Discussion

The mammary gland’s ability to produce milk, which holds indispensable significance for neonatal well-being and human health, underscores the importance of maintaining optimal mammary gland health in cows. While the vital regulatory role of lncRNAs has been extensively studied in mice and humans, their involvement in bovine mammary gland biology remains a developing field [41]. Interestingly, lncRNAs have been found to exert a critical influence on mammary gland development and breast cancer biology [42]. In recent years, there has been a notable surge in studies exploring the role of lncRNAs in bovine mammary gland tissues, further expanding our understanding of their intricate functions in this context.

Several studies have shed light on the role of lncRNAs in mammary gland development and function in various species. Previously, the research focused on water buffalo, identifying 7962 lncRNA genes and highlighting the significantly different expression of lnc-bbug14207 between early- and late-lactation stages, suggesting its potential involvement in regulating neighboring protein-coding genes critical for mammary gland development [41]. Another investigation using high-throughput single-cell RNA-seq data explored the hierarchical model of mammary epithelium, revealing that lncRNAs exhibited lineage-specific expression and formed a triplex structure with DNA to regulate downstream lineage-specific marker genes [43]. In a study involving sheep, differentially expressed lncRNAs and mRNAs were identified in the pituitary gland between immature lncRNAs in male reproduction [44]. Additionally, an examination of lncRNAs in swine identified differentially expressed lncRNAs involved in mammary gland development, lactation, milk composition metabolism, and colostrum function, suggesting their potential role in the occurrence processes of parturition and colostrum [45]. These studies collectively contribute to our understanding of the diverse roles of lncRNAs in mammary gland biology across different species.

In cows, about23,515 lncRNA transcripts have been reported (http://www.noncode.org/index.php). Ji et al. identified 2381 potential lncRNAs in different lactation stages of dairy goats [46]. Zheng et al. were the first to systematically identify the lncRNAs using RNA-seq of the bovine mammary gland at different lactation stages, and 1657 lncRNAs transcripts from 1181 candidate lncRNA loci were finally obtained [40]. The cow is more susceptible to mastitis at both prenatal and postpartum stages, and mastitis has a relatively high incidence in dairy cows; once the cow is infected with the disease, it will not only cause adverse effects on the amount and quality of milk production, but also seriously harm the cow’s health [47]. Chen et al. found 19 differentially expressed lncRNAs from Staphylococcus aureus infected and non-infected cow mammary epithelial cell lines, and these lncRNAs were involved in inflammation-related signaling pathways (the Notch signaling pathway, TNF signaling pathway, and NF-κB signaling pathway) [47]. In summary, these studies demonstrate that lncRNAs have regulatory effects on different lactation stages and mammary gland health in dairy cows.

In the present study, we used a high-throughput sequencing method to detect lncRNAs in the early-lactation stage and non-lactation stage of Holstein cows, and to screen the important lncRNAs in the mammary gland at these two stages. Using the methods of transcript splicing and transcript comparison, as well as coding potential screening, a total of 1905 lncRNAs were identified, of which 96 lncRNAs were differentially expressed, including 83 upregulated and 53 downregulated. And these lncRNAs identified in this study are of a high quality: 57.3% of the predicted lncRNAs are ≥500 bp, and 12.9% are ≥2000 bp. A total of 612 lncRNAs are intronic lncRNAs, which are the largest of the four types of lncRNAs. In addition, the exon number of lncRNAs ranged from 2 to 10, but only 1 lncRNA had 10 exons.

The function of lncRNAs has not been fully clarified. They can interact with coding genes in cis and trans ways to realize their functions [21]. GO and KEGG pathway analysis were performed after we obtained lncRNAs’ cis and trans target genes to predict the related functions of target genes. Target gene GO analysis results showed that target genes were mainly involved in the developmental process, biological regulation, and regulation of the biological process, single-organism process, metabolic process, and immune system process. Cellular components were mainly related to the extracellular region, extracellular matrix component, and cell junction. In terms of molecular function, they were significantly correlated with nucleic acid-binding transcription factor activity, binding, and structural molecule activity. KEGG pathway analysis showed that target genes mainly enriched in a metabolic pathway, hematopoietic cell lineage pathway, and PI3K-Akt signaling pathway [48].

The PI3K Akt signaling pathway has emerged as a pivotal pathway involved in the growth, proliferation, and survival of mammalian cells, including mammary epithelial cells. Recent studies have also linked this pathway to the regulation of inflammatory responses [49,50]. In the context of this study, the lncRNAs TCONS_00010180 was found to exert its regulatory influence on the CD36 gene in a cis manner. CD36 is a membrane glycoprotein present on certain epithelial cells and is known to contribute to inflammatory responses. Furthermore, the lncRNAs TCONS_00023829 and TCONS_00041715 were found to regulate the trans-target genes TLR2 and MAPK4, respectively. These genes, along with CD36, are involved in multiple pathways associated with inflammatory responses, including ECM-receptor interaction, AMPK signaling pathway, NF-κB signaling pathway, and the well-known PI3K-Akt signaling pathway. Notably, these pathways play crucial roles in the regulation of cytokine production, immune responses, and inflammatory processes, all of which have been implicated in the occurrence, development, and regulation of mastitis. Moreover, the activation of TLR4 by LPS in mammary epithelial cells can initiate innate immune and inflammatory responses through the activation of NF-κB and MAPK pathways, leading to the expression of pro-inflammatory cytokines such as IL-6 and TNF-α.

Overall, most of the pathways enriched by the target genes of the differentially expressed lncRNAs in this study were associated with the development of mastitis. We speculated that lncRNA TCONS_00010180, TCONS_00023829, and TCONS_00041715 might affect mammary gland development and lactation by regulating the expression of CD36 in the bovine mammary gland. Therefore, we will subsequently focus on lncRNA TCONS_00010180, TCONS_00023829, and TCONS_00041715 and their target gene CD36, TLR2, and MAPK4, respectively, in an in-depth study.

We do, however, recognize that our study design has certain inherent limitations. Despite being a typical practice in transcriptome investigations, using three biological replicates per group may not adequately reflect the heterogeneity present within individual animals, thus impacting the discovery of low-abundance lncRNAs. Future research should take into account bigger sample sizes to solve this issue and allow for a more thorough analysis of lncRNA expression dynamics. Furthermore, we acknowledge that our study did not account for possible dietary impacts, which might have affected the patterns of lncRNA expression that were found. Future research should use controlled dietary interventions to further understand how nutrition affects lncRNA dynamics in relation to mammary gland growth and function.

## 4. Materials and Methods

### 4.1. Animal Ethics Statement

In all experiments, the Ministry of Science and Technology of the People’s Republic of China approved the use and care of experimental animals (approval number 2006-398). Following the welfare ethics of experimental animals, mammary gland tissue samples were collected, and a production license for experimental animals was obtained (SYDW-2019005). The experimentation was also approved by Yangzhou University, Yangzhou, China.

### 4.2. Animal Sample Collection

In this study, mammary gland tissues were sampled from three Holstein cows in early lactation (*n* = 3, 30 days postpartum) and non-lactation (*n* = 3, 315 days postpartum) on a large dairy farm in Jiangsu province. All cows were fed with a standard diet, according to their requirements. The composition of experimental diets is listed in Table 3. Before collecting tissue samples from mammary glands of lactating cows free of mastitis, milk was completely extruded from the glands. The biopsy sampling methodology is detailed by Li et al. [51]. In brief, cow hair was removed from sample sites, and then the skin was disinfected with 75% ethanol and anesthetized subcutaneously with 1 mL procaine.

After a 1.5 cm incision was made at the sampling site, sterile scissors and forceps were used to remove the connective tissues to expose the parenchyma. Then, mammary gland tissue biopsies (1–2 g) were harvested, washed with PBS buffer (Invitrogen, Carlsbad, CA, USA), and immediately frozen in liquid nitrogen until the RNA was isolated. Finally, to clamp the skin incision closed, an 11 mm wound clip was used, as well as povidone iodide cream evenly applied to the incision.

### 4.3. RNA Extraction and Sequencing

The mammary gland tissue was treated with TRIzol reagent (Invitrogen, Carlsbad, CA, USA), and the total RNA was then extracted using the RNAprep Pure Tissue Kit (Tiangen, RNAprep Pure Tissue Kit, Beijing, China). The quantity of total RNA was greater than 400 ng/L, and the 260/280 requirement was 1.9~2.0. A sequencing library was constructed, and genomic sequence mapping and analysis were performed. Ribosomal RNA was removed from the mammary tissue RNA samples using a transcriptome isolation kit (Ribominus Bacteria 2.0, Thermo Fisher Scientific, Waltham, MA, USA). The remaining RNA was paired-end sequenced using an Illumina HiSeq Xten (Illumina Inc., San Diego, CA, USA) from Shanghai Personal Biotechnology Company, Ltd. (Shanghai, China). Through the Illumina platform, a large amount of sample paired-end sequencing data was obtained. Given the impact of data error rate on the results, we employed Trimmomatic [52] software (v0.36) to preprocess the raw data for quality and to statistically summarize the number of reads throughout the quality control process.

### 4.4. Identification and Expression Level of lncRNAs

The String Tie [53] software (v1.3.3b) is used to reconstruct the transcripts in the samples based on the probability model of the comparison results of each sample, and the chain direction of the transcripts can be accurately determined for the data of the chain-specific library. For multi-sample projects, String Tie is used to combine the reconstructed transcripts of each sample to produce a collection of transcripts that represent the transcription of the samples of the project: (1) The spliced transcripts were linked and examined with reference transcripts to screen out new transcripts with known coding transcripts or known loci. (2) The transcripts obtained from (1) screening were screened according to the length of ≥200 bp and the number of exons ≥2. (3) We predicted and analyzed the coding ability of the transcripts screened, and screened out the transcripts with coding potential. CPC [54], CNCI [55], Pfam [56], and PLEK [57] were used. The obtained lncRNA sequences were characterized. (4) For species with known lncRNA, we compared the obtained lncRNA sequence in (3) with known lncRNA, using blast software (https://blast.ncbi.nlm.nih.gov/Blast.cgi, accessed on 22 August 2023) to screen out duplicate sequences. After merging with the known lncRNA sequence, quantitative analysis was performed.

### 4.5. Analysis of the Co-Expression of Differentially Expressed mRNAs and lncRNAs

The number of counts for each lncRNA across samples were normalized using the DEseq [58] software (v1.18.0) (BaseMean value was used to estimate the expression), the fold difference was calculated, the significance of the read count difference was tested using NB (using the negative binomial distribution test), and then the differential genes were screened based on the fold difference and significance of the read-count-difference test results. Differences were automatically filtered on *p* < 0.05 and fold change > 2.

### 4.6. Prediction of the Target Gene and Functional Analysis

The intersection of co-expressed genes with differentially expressed lncRNAs (calculated by Pearson correlation) was performed for all coding genes within 100 kbp upstream and downstream. In order to identify target sequences, we selected lncRNAs and mRNAs located on different chromosomes and extracted candidate sequences based on differential co-expression results [59]. We used the RNA interaction software RIsearch-2.0 to predict the binding of candidate lncRNA and mRNAs at the nucleic acid level, according to the following conditions: it is likely that the screened interacting lncRNA and mRNA may exist in direct regulations, which are called trans-target genes, if the number of bases directly interacting is no fewer than 10 and the free energy of base binding is no more than −50 for the screening conditions. Gene Ontology (GO) enrichment and the KEGG pathway analysis were used to investigate the main function of the target genes of the differentially expressed lncRNAs using the DAVID tool (https://david.ncifcrf.gov, accessed on 2 July 2023) and Kyoto Encyclopedia of Genes and Genomes (KEGG, http://www.kegg.jp/, accessed on 2 July 2023) pathway analyses.

### 4.7. Statistical Analysis

A data set was collected and processed, and the experimental diagrams were prepared with an MS Excel sheet. The prediction of cis- and trans-target genes was based on the possible co-expression relationship between lncRNA and mRNA. The GO and KEGG analysis of DElncRNA target genes was carried out using DAVID (https://david.ncifcrf.gov/, accessed on 2 July 2023). Pearson’s correlation test was used to calculate the expression correlation between the two, according to the differential lncRNA and mRNA expression data, and relationship pairs with a correlation coefficient not lower than 0.8 and a *p*-value less than or equal to 0.05 were selected, and considered to have a co-expression relationship.

## 5. Conclusions

In this study, RNA-seq analysis was conducted to identify and characterize lncRNAs in Holstein cow mammary tissues during the early-lactation and non-lactation stages. The findings revealed a total of 1095 lncRNAs, with 136 demonstrating differential expressions between the two stages. These differentially expressed lncRNAs, with an average length of 1065 base pairs, were found primarily on chromosomes 1–21. Intronic lncRNAs emerged as the most common subtype among the lncRNAs identified. Pathway analysis was performed to understand the functional implications of these differentially expressed lncRNAs. The analysis showed that these lncRNAs were particularly enriched in signaling pathways such as TLR2, PI3K-Akt, and MAPK4. This suggests that they could be involved in important signaling cascades and regulatory processes related to immune responses, cellular growth, and intracellular signaling. This study lays a solid foundation for future research on the functional roles of lncRNAs by elucidating the expression profiles and characteristics of lncRNAs during different lactation stages in Holstein cows. Despite the fact that our results shed light on the potential regulatory functions of lncRNAs, it is important to be aware of the study’s drawbacks, such as the small sample size and potential dietary impacts. The complex roles lncRNAs play in bovine mammary gland development and function will be better understood in future studies with bigger sample sizes and controlled dietary treatments.

## Figures and Tables

**Table 1:** LncRNA sequence information statistics.

Term	All ^1^	≥200 bp ^2^	≥500 bp ^3^	≥1000 bp ^4^	N50 ^5^	Total_Length ^6^	Max_Length ^7^	Min_Length ^8^	Average_Length ^9^
lncRNA	1905	1905	1091	559	1833	2,028,835	18449	201	1065.01

(1) All: total number of lncRNAs; (2) ≥200 bp: length ≥200 bp number of lncRNAs; (3) ≥500 bp: length ≥500 bp number of lncRNAs; (4) ≥1000 bp: length ≥1000 bp number of lncRNAs; (5) N50: add the sequences in order from long to short; when the added length reaches half of the total length of the sequence, the last added sequence length is N50; (6) Total Length: the total number of bases of lncRNA; (7) Max Length: the longest lncRNA length; (8) Min Length: the shortest lncRNA length; (9) Average Length: Average length.

**Table 2:** Differentially expressed lncRNAs in mammary tissue from Holstein cows at early lactation and non-lactation.

lncRNAs ID	Fold Change	log_2_Fold Change	*p*-Value	Up/ Down Change ^2^	Gene ID	BaseMean Control ^3^ 315 d	BaseMean Case ^4^ 30 d
TCONS_00000127	0.3159	−1.662	0.0484	Down	XLOC_000078	46.65	14.73
TCONS_00000573	0.2427	−2.042	0.00009	Down	XLOC_000422	332.4	80.71
TCONS_00000892	7.237	2.855	0.045	Up	XLOC_000639	9.581	69.34
TCONS_00001928	32.77	5.034	0.0055	Up	XLOC_001382	5.348	175.29
TCONS_00002045	2.2491	1.169	0.0164	Up	XLOC_001477	243.6	548.04
TCONS_00004577	2.4189	1.27	0.0203	Up	XLOC_003131 XLOC_003235	177.6	429.74
TCONS_00004705	Inf ^1^	Inf	0.0038	Up		0	16.12
TCONS_00006010	6.1316	2.6162	0.0020	Up	XLOC_004109	11.86	72.74
TCONS_00007339	0.4084	−1.291	0.00090	Down	XLOC_004984	912.2	372.57
TCONS_00007734	2.306	1.2059	0.0090	Up	XLOC_005241	149.6	345.11
TCONS_00007858	0.2149	−2.217	0.0484	Down	XLOC_005316	88.74	19.07
TCONS_00007979	Inf	Inf	0.04212	Up	XLOC_005396	0	10.26
TCONS_00008771	81.063	6.340	0.0278	Up	XLOC_006002	1.640	132.96
TCONS_00008773	33.52	5.067	0.0098	Up	XLOC_006003	35.85	1201.8
TCONS_00010180	19.627	4.294	4.2221	Up	XLOC_007129	32.80	643.83
TCONS_00010615	Inf	Inf	0.041	Up	XLOC_007443	0	8.11
TCONS_00010780	3.5306	1.819	0.013	Up	XLOC_007571	16.12	56.94
TCONS_00011358	2.1697	1.117	0.0048	Up	XLOC_008032	35,441.9	76,899.5
TCONS_00012030	0.0753	−3.729	0.047	Down	XLOC_008500	14.55	1.097
TCONS_00012647	0.2409	−2.053	0.043	Down	XLOC_008902	41.19	9.925
TCONS_00012998	10.057	3.330	0.0454	Up	XLOC_009211	4.028	40.51
TCONS_00013114	12.2699	3.6170	0.0206	Up	XLOC_009282	12.10	148.56
TCONS_00014214	120.43	6.912	0.0002	Up	XLOC_010015	0.556	67.073
TCONS_00014380	0.447	−1.161	0.0309	Down	XLOC_010130	250.66	112.06
TCONS_00017362	5.416	2.4372	0.0081	Up	XLOC_012269	45.81	248.16
TCONS_00017554	6.236	2.6408	0.00088	Up	XLOC_012384	65.91	411.13
TCONS_00018805	5.469	2.451	0.0030	Up	XLOC_013313	10.77	58.95
TCONS_00019123	2.2016	1.1385	0.016	Up	XLOC_013518	2072.96	4563.8
TCONS_00020026	0.499	−1.001	0.045	Down	XLOC_014135	436.85	218.17
TCONS_00020595	2.532	1.340	0.032	Up	XLOC_014531	36.46	92.34
TCONS_00021189	44.226	5.4668	0.010	Up	XLOC_014971	0.526	23.28
TCONS_00021586	0.337	−1.567	0.00003	Down	XLOC_015283	149,600.4	50,464.8
TCONS_00022016	Inf	Inf	0.007	Up	XLOC_015571	0	12.20
TCONS_00022067	0.0338	−4.88	0.013	Down	XLOC_015611	35.93	1.215
TCONS_00023829	10.066	3.331	0.0035	Up	XLOC_016897	44.15	444.5
TCONS_00024356	5.110	2.353	0.039	Up	XLOC_017317	31.69	161.98
TCONS_00024512	110.983	6.794	2.214	Up	XLOC_017449	1.104	122.56
TCONS_00024513	44.96	5.490	0.003	Up	XLOC_017449	1.842	82.85
TCONS_00024834	5.4621	2.449	0.0009	Up	XLOC_017667	51,842.6	283,172.0
TCONS_00024835	0.490	−1.0277	0.004	Down	XLOC_017667	205,345.5	100,714.3
TCONS_00024837	7.4177	2.890	0.0009	Up	XLOC_017667	6292.51	46,676.4
TCONS_00024971	0.2988	−1.742	0.020	Down	XLOC_017757	78.287	23.39
TCONS_00024975	0.05063	−4.303	0.018	Down	XLOC_017757	33.67	1.704
TCONS_00024978	0.0286	−5.123	7.275	Down	XLOC_017757	193.08	5.537
TCONS_00024979	0	Inf	0.012	Down	XLOC_017757	12.54	0
TCONS_00024982	0.1028	−3.281	0.010	Down	XLOC_017757	169.87	17.47
TCONS_00025065	27.54	4.783	0.012	Up	XLOC_017811	20.204	556.5
TCONS_00025067	12.063	3.592	0.037	Up	XLOC_017811	2.43	29.33
TCONS_00026548	78.218	6.289	0.016	Up	XLOC_018845	0.83	65.34
TCONS_00026777	2.355	1.235	0.011	Up	XLOC_019017	87.54	206.19
TCONS_00026788	69.85	6.289	2.475	Up	XLOC_019024	5.409	377.87
TCONS_00027914	0	Inf	0.016	Down	XLOC_019812	21.38	0
TCONS_00028242	0	Inf	0.023	Down	XLOC_020028	12.286	0
TCONS_00028308	15.025	3.909	0.035	Up	XLOC_020075	39.73	597.08
TCONS_00028334	2.2416	1.164	0.011	Up	XLOC_020089	98.86	221.61
TCONS_00028551	10.535	3.397	0.009	Up	XLOC_020238	2.506	26.40
TCONS_00028770	2.270	1.183	0.007	Up	XLOC_020344	173.28	393.48
TCONS_00028872	Inf	Inf	0.024	Up	XLOC_020430	0	13.47
TCONS_00029140	7.055	2.818	0.034	Up	XLOC_020626	11.87	83.7
TCONS_00029471	4.2468	2.086	0.009	Up	XLOC_020853	14.83	62.99

^1^ Inf = infinity. ^2^ The difference in the expression of a specific lncRNA in the mammary tissue during the non-lactating period, compared with peak lactation. ^3^ BaseMean is used to homogenize the gene expression in both the total sample and the single sample in the DEGSeq R package. BaseMean Control is the BaseMean value of mammary tissue with peak lactation. ^4^ BaseMean Case is the BaseMean value of mammary tissue in the non-lactating period. (Specific data for Differentially expressed lncRNAs are presented in the Supplementary Materials, Table S1).

**Table 3:** Diet composition and nutritional values given to experimental cattle in our study.

Items	Content (%)
Alfalfa hay	8.39
Oat hay	6.43
Whole corn silage	52.18
Corn	8.1
Soya bean meal	10.8
Cotton seed meal	6.8
DDGS ^1^	4.8
Premix	2.5
Total	100

## Data Availability

Data presented in this study are available upon request from the corresponding author.

## Supplementary Materials

The following supporting information can be downloaded at: https://www.mdpi.com/article/10.3390/ijms241713585/s1.

## Abbreviations

lncRNAs	long non-coding RNAs
ncRNAs	non-coding RNA
MAPK4	Mitogen-activated protein kinases4
TLR2	Toll-like receptor 2
AMPK	activated protein kinase
KEGG	Kyoto Encyclopedia of Genes and Genomes
GO	Gene Ontology
PCA	principal component analysis
WTSS	whole transcriptome sequencing studies
mRNAs	protein-coding messenger RNAs
